# The effects of genetic polymorphisms of APOE on circulating lipid levels in middle-aged and elderly chinese Fujian Han population: toward age- and sex-personalized management

**DOI:** 10.1186/s12944-021-01587-6

**Published:** 2021-11-08

**Authors:** Xiaofeng Liu, Qingwen Lin, Kengna Fan, Minjie Tang, Weiqing Zhang, Bin Yang, Qishui Ou

**Affiliations:** 1 Department of Laboratory Medicine, the First Affiliated Hospital of Fujian Medical University, 20 Chazhong Road, 350005 Fuzhou, China; 2 Fujian Key Laboratory of Laboratory Medicine, No.20 Chazhong Road, 20 Chazhong Road, 350005 Fuzhou, China; 3 Gene Diagnostic Laboratory, the First Affiliated Hospital of Fujian Medical University, 350005 Fuzhou, China

**Keywords:** Apolipoprotein E, Genetic polymorphism, Serum lipids, Gender, Geriatrics

## Abstract

**Background:**

Increased evidence has reported the association of genetic polymorphisms of Apolipoprotein E (APOE) with serum lipids. However, few studies have explored the combined effects of APOE, gender, and age.

**Methods:**

A total of 1,419 middle-aged and elderly subjects were randomly selected and studied. The APOE genotypes and the serum lipids were detected. The effects of APOE, gender, and age on serum lipids were preliminarily observed in general. The subjects were then divided into the middle-aged group (40–64 years old) and the elderly group (≥ 65 years old), for both males and females, to explore the combined effects of the APOE, gender, and age on serum lipids. Finally, a multivariate logistic regression model was used to evaluate the associations between the APOE allele carriers and the at-risk levels of dyslipidemia.

**Results:**

The serum TC, LDL-C, and ApoB in the ε2 carriers were lower than the ε3 carriers (all *P* < 0.05), and there was no significant difference in the ε4 carriers compared to the ε3 carriers in general (all *P* > 0.05). The serum LDL-C and ApoB of the ε2 carriers were lower than the noncarriers in the middle-aged and elderly males (all *P* < 0.05). The serum TC in the ε2 carriers was lower than the noncarriers only in middle-aged males (*P* < 0.05). As to the levels of serum HDL-C and ApoA1, the ε2 carriers were higher than the noncarriers in middle-aged females (all *P* < 0.05), and the ε4 carriers were lower than noncarriers in middle-aged males (*P* < 0.05). Especially, the serum TG in the ε4 carriers was significantly higher than the noncarriers in elderly females. The logistic regression analysis indicated that the ε2 carriers were less likely to have at-risk levels of high LDL-C in middle-aged and elderly males (all *P* < 0.05) versus low HDL-C in middle-aged females (*P* < 0.05). In contrast, the ε4 carriers were more likely to have at-risk levels of high TG in elderly females (*P* < 0.05).

**Conclusions:**

The effects of the genetic polymorphisms of APOE on the serum lipids were both gender- and age-dependent in the middle-aged and elderly Chinese Fujian Han population.

## Introduction

With the acceleration of China’s aging population, the health of the elderly has attracted more and more attention [1]. The aging population will endure more chronic medical conditions such as cardiovascular disease, diabetes mellitus, and stroke, which have become the major morbidity and mortality [2]. Dyslipidemia is a well-established risk factor of these chronic diseases [3, 4]; however, our understanding of age-related dyslipidemia is still incomplete.

Serum lipids are strongly influenced by smoking, diet, levels of physical activity, and other lifestyles choices. However, the studies from twins and families suggest that the genetic factors are involved in the variation of serum lipids [5, 6]. Apolipoprotein E (APOE), as a critical apolipoprotein, plays a central role in cholesterol metabolism and transport. The APOE gene, as a commonly investigated polymorphic genetic marker, encoded three major alleles: ε2, ε3, and ε4, composed of six genotypes: ε3/ε3, ε3/ε4, ε2/ε3, ε2/ε4, ε2/ε2, and ε4/ε4. The ε3/ε3 genotype was considered a “wild type” because it is the widest distribution of the populations [7]. Human ApoE is a 34-kDa protein comprised of three common isoforms (ApoE2, ApoE3, and ApoE4) [8]. Previous studies have shown that serum lipids are under genetic influence by the APOE polymorphisms [9–11]. However, most studies were conducted in the general population. Few studies were performed on the middle-aged and elderly population, particularly in the Chinese Han population of Fujian Province located on the southeast coast of China.

Additionally, it has been reported that gender is involved in lipid and lipoprotein metabolism [12, 13]. The changes in serum lipid levels exist in gender differences during aging [14]. Increased evidence has identified the association between sex-specific genetic and serum lipids [15, 16]. The combined effects of APOE genetic polymorphism, sex and age on the serum lipids during aging were less reported, which would provide some valuable insight on the individualized lipid management for the aging population. This study aimed to explore the effects of APOE genetic polymorphisms on serum lipids in the middle-aged and elderly Chinese Han population of Fujian Province and observed whether these effects exhibit gender-and age-specificity during aging.

## Methods

### Study population

In the present study, 1503 subjects located in Fujian Province were enrolled initially. 46 subjects were excluded because the clinical data were incomplete, and 38 subjects were excluded due to insufficient biological specimen sampling. Finally, we enrolled 1419 middle-aged and elderly subjects (aged 40 years and above) who had received routine health check-ups at the First Affiliated Hospital of Fujian Medical University (Fuzhou, China) between June 2017 and June 2021. All participants were Chinese with Han ethnicity, born in Fujian, and lived in Fujian Province (China). Those who were not born in Fujian and just lived in Fujian Province were currently excluded. The others suffering from severe cardiac diseases, kidney and pulmonary diseases, infective diseases, tumors, diabetes mellitus, chronic liver disease, and subjects with shorter survival time were excluded. The study was performed following the Declaration of Helsinki and was approved by the ethics committees of the First Affiliated Hospital of Fujian Medical University (No, MRCTA, ECFAH of FMU,2018[046]). The written informed consent was obtained from the participants or their surrogates before the study.

### Serum lipid parameters measurement

Peripheral venous blood (3~5mL) was collected from all subjects after at least 12 h of fasting. Centrifuged at 1,500 g for 10 min for getting serum as soon as possible, then stored at -80 °C until used. An automatic biochemistry analyzer (Siemens, ADVIA2400) was used to examine the levels of serum lipids, including serum high-density lipoprotein cholesterol (HDL-C), low-density lipoprotein cholesterol (LDL-C), total cholesterol (TC), triglyceride (TG), ApolipoproteinA1 (ApoA1), and Apolipoprotein B (ApoB).

### Apolipoprotein E genotype analysis

The APOE genotypes were determined according to our previous methods [17]. Briefly, the genomic DNA was extracted from peripheral blood leukocytes by a DNA extraction kit (Qiagen, Hamburg, Germany). The extracted DNA quality was evaluated by a NanoDrop 1000 spectrophotometer (Thermo Fisher, Waltham, MA, USA). The genotype of APOE was detected using a commercial DNA Chip Detection kit (Zhuhai Sinochips Bioscience Co., Ltd, Guangdong, China), which has been approved by the CFDA (China Food and Drug Administration), according to the manufacturer’s instructions. Then, the human APOE fragments, including two SNP´s (rs429358 (Cys112Arg) and rs7412 (Arg158Cys), were amplified by PCR, and the PCR products were hybridized through the specific probe. The hybrid product was colored by the chromogenic agent, and the images in the chip were scanned and the genotype was determined. At the same time, the APOE genotype was further confirmed by Sanger sequencing and blasted in the GenBank [18]. The primers for the Sanger sequencing are as follows: forward primer (5’->3’), GACCATGAAGGAGTTGAAGGCCTAC; reverse primer (5’->3’), CTCGCGGGCCCCGGCCTGGTA.

### Statistical analysis

Data analysis was performed using the SPSS 20.0 software (Chicago, USA). For continuous variables, the data were expressed as the mean ± standard deviation (SD), and the categorical variables were represented as a number (%). The Hardy-Weinberg equilibrium in all participants was examined. Using the independent t-test, one-way ANOVA, or the Chi-square test to compare the outcomes. To evaluate the combined effects of APOE genotype, gender and age on serum lipids, we used the logistic regression models to analyze the associations between APOE alleles carrier and at-risk levels of serum dyslipidemia. The BMI, smoking, drinking, education, and physical activity were critically adjusted as confounding factors. The statistical significance was set as *P*< 0.05, and all tests were two-tailed.

## Results

### General characteristics of the participants

The characteristics of the studied subjects and serum lipids are shown in Table 1. There were 795 males (56.03 %) and 624 females (43.97 %), and the average age was 59.18 ± 12.53 years and ranged from 40 to 91 years. The average BMI (body mass index) of all participants was 24.96 ± 3.34, for males was 24.92 ± 3.62, and for females was 25.45 ± 4.02. Meanwhile, 12.94 % of the subjects reported having the habit of smoking, including 31.71 % for males and 0.94 % for females (*P* < 0.05). Additionally, 18.75 % of the subjects reported having the habit of drinking, including 32.06 % for males and 9.84 % for females (*P* < 0.05), and 36.91 % of the subjects reported no exercise, including 37.56 % for males and 36.82 % for females (*P* > 0.05).

**Table 1:** General characteristics and serum lipids of the study participants

	All(*n*=1419)	Male(*n*=795)	Female(*n*=624)	*P* value
**Age(years)**	59.18±12.53	58.41±12.61	59.57±12.37	0.282
**Sex, n (%)**	—	795(56.0)	624(44.0)	—
**BMI (kg/m**^**2**^**)**	24.96±3.34	24.92±3.62	25.45±4.02	0.043
**Smoking n (%)**	258(18.18)	252(31.7)	6(0.9)	<0.001
**Drinking n (%)** **No-Exercise n (%)**	316(22.26) 528(37.2)	255(32.1) 298(37.5)	61(9.8) 230(36.8)	<0.001 0.703
**TC (m mol/L)**	5.02±0.99	4.94±0.96	5.16±1.02^*^	<0.001
**TG (m mol/L)**	1.46±0.96	1.56±1.09	1.32±0.68^*^	<0.001
**HDL-C (m mol/L)**	1.35±0.38	1.25±0.29	1.48±0.44^*^	<0.001
**LDL-C (m mol/L)**	3.18±0.92	3.15±0.92	3.24±0.89	0.073
**ApoA1(m mol/L)**	1.38±0.17	1.35±0.15	1.43±0.19^*^	<0.001
**ApoB (m mol/L)**	1.01±0.33	1.04±0.35	1.01±0.39	0.227
**A1/B**	1.37±0.56	1.30±0.53	1.41±0.56^*^	0.001

Data are expressed as the means ± standard deviation, except for sex, smoking, drinking, and No-exercise, which is shown as the number (%). BMI, Body mass index, *TC* Total cholesterol, *TG* Triglyceride, *HDL-C* High density lipoprotein cholesterol, *LDL-C* Low density lipoprotein cholesterol. *ApoA1* apolipoproteinA1, *ApoB* apolipoprotein B. A1/B, the ratio of ApoA1 and ApoB

*P*-value, 2-sample independent t-test, or chi-squared test as appropriate. *P<0.05, compared with males

As to the profiles of serum lipids, the serum TC, HDL-C, ApoA1, and the ratio of ApoA1 and ApoB (referred to as A1/B) in females were higher than males (all *P* < 0.05), while the serum TG in males was significantly higher than in females (*P* < 0.05). No significant differences were found in the serum LDL-C and ApoB between males and females (all *P* > 0.05). These data indicated a significant sex difference of serum lipids in the middle-aged and elderly Chinese Han population in Fujian Province.

### APOE allele frequencies and genotypes

First, the Hardy-Weinberg equilibrium in all subjects was estimated, and the APOE allelic frequency was following Hardy-Weinberg equilibrium (*P* = 0.17 > 0.05). As shown in Table 2, we identified the six common genotypes of APOE, and the genotype ε3/ε3 was greatest in all of the participants, followed by ε3/ε4, ε2/ε3, ε2/ε4, ε4/ε4, and ε2/ε2, respectively. Similarly, the carriers of ε3 were greatest (82.91 %), followed by the carriers of ε4 (9.16 %) and ε2 (7.93 %). Meanwhile, there were no significant differences in the APOE allele frequencies and genotypes between the males and females (all *P*>0.05), which was in line with the previous studies in the Asian population [19].

**Table 2:** Allele frequencies and distribution of APOE genotypes in middle-aged and elderly Fujian Han population

Genotype /Allele	All(*n*=1419)		Male (*n*=795)		Female (*n*=624)		*P* value
	**No**	**%**	**No**	**%**	**No**	**%**	
**ε2/ε2**	11	0.78	7	0.88	4	0.64	0.861
**ε2/ε3**	176	12.40	92	11.57	84	13.46	0.342
**ε2/ε4**	27	1.90	16	2.01	11	1.76	0.678
**ε3/ε3**	989	69.70	563	70.82	426	68.27	0.407
**ε3/ε4**	199	14.02	109	13.71	90	14.42	0.748
**ε4/ε4**	17	1.20	8	1.01	9	1.44	0.787
**ε2**	225	7.93	122	7.67	103	8.25	0.561
**ε3**	2353	82.91	1327	83.46	1026	82.21	0.526
**ε4**	260	9.16	141	8.87	119	9.54	0.776

The ε2 carriers included the genotypes ε2/ε2, ε2/ε3 and ε2/ε4; the ε3 carriers included ε3/ε3; the ε4 carriers included ε3/ε4 and ε4/ε4. The categorical variables were carried out by the chi-squared test

### Serum lipids according to APOE genetic polymorphism

**Fig. 1:**
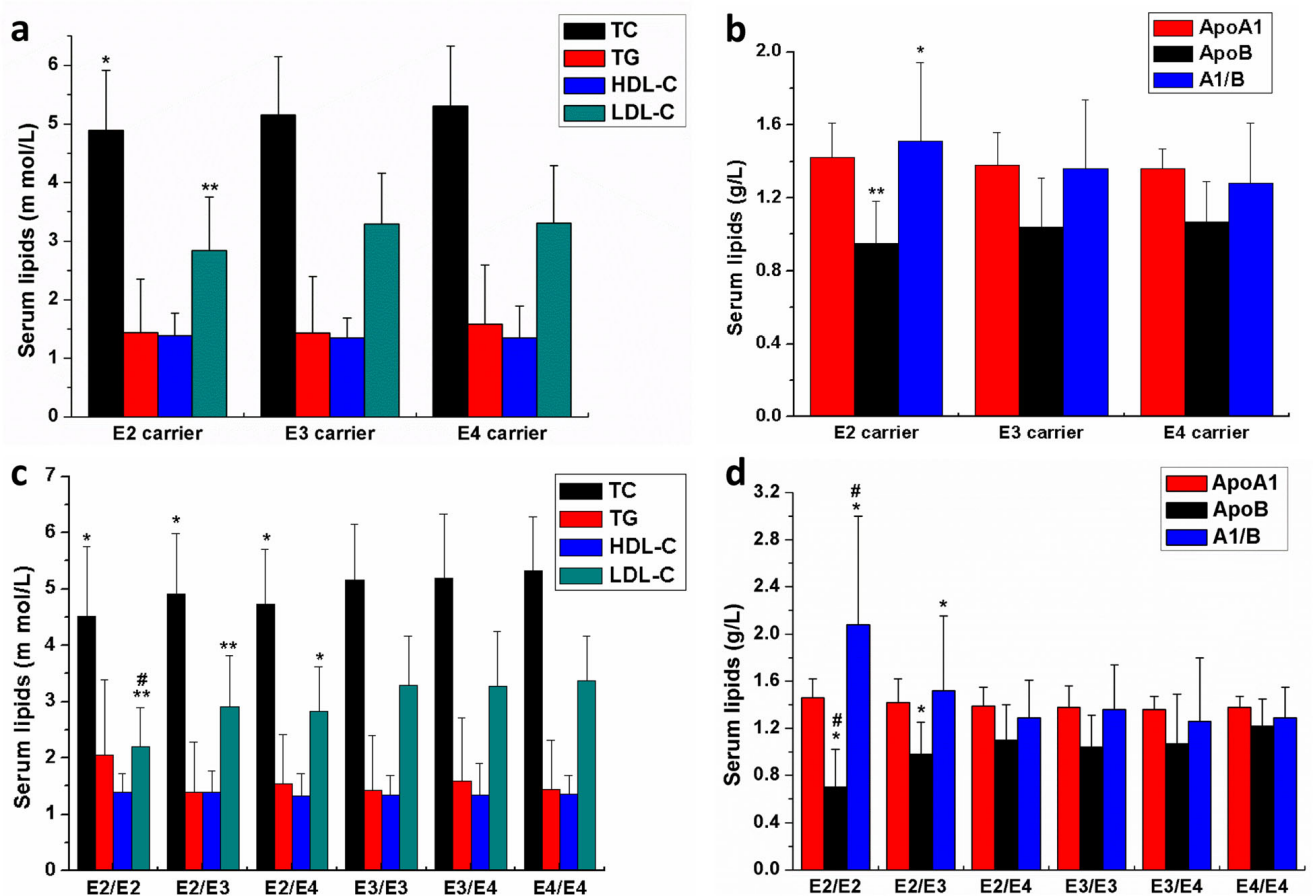
The influences of APOE genetic polymorphisms on serum lipids of the middle-aged and elderly Fujian Han population in general. The effects of APOE allele carriers on serum lipids of the middle-aged and elderly Fujian Han population in general (**A** and **B**); The effects of APOE genotypes on serum lipids of the middle-aged and elderly Fujian Han population in general (**C** and **D**). TC=Total cholesterol, TG=Triglyceride, HDL-C=High density lipoprotein cholesterol, LDL-C=Low density lipoprotein cholesterol. ApoA1=apolipoproteinA1, ApoB=apolipoprotein B. *P<0.05, **P<0.01, ε2 carriers VS noncarriers (ε3 carriers and ε4 carriers)

To preliminarily evaluate the effects of APOE on serum lipid levels, we compared the levels of serum lipids according to the APOE carriers and genotypes[20]. As shown in Table 3, the serum TC, LDL-C, and ApoB were lower in the ε2 carriers (ε2/ε2, ε2/ε3, and ε2/ε4) compared to the ε3 carriers (ε3/ε3) and ε4 carriers (ε3/ε4 and ε4/ε4; all *P* < 0.05, Fig. 1A and B), and serum A1/B of the ε2 carriers was higher than the carriers of ε3 and ε4 carriers (*P* < 0.05, Fig. 1B).

**Table 3:** The association of APOE genetic polymorphisms with serum lipids in middle-aged and elderly Fujian Han population

Allele/ Genotype	N	TC (m mol/L)	TG (m mol/L)	HDL-C (m mol/L)	LDL-C (m mol/L)	ApoA1 (g/L)	ApoB (g/L)	A1/B
**ε2 carrier**	214	4.89±1.02^*^	1.44±0.91	1.39±0.38	2.84±0.91^**^	1.42±0.19	0.95±0.23^**^	1.51±0.43^*^
**ε3 carrier**	989	5.16±0.99	1.43±0.97	1.35±0.34	3.29±0.87	1.38±0.18	1.04±0.27	1.36±0.38
**ε4 carrier**	216	5.31±1.02	1.58±1.01	1.35±0.54	3.31±0.98	1.36±0.11	1.07±0.22	1.28±0.33
**ε2/ε2**	11	4.51±1.24^*^	2.05±1.35	1.39±0.33	2.20±0.69^** #^	1.46±0.16	0.70±0.32^* #^	2.08±0.92^* #^
**ε2/ε3**	176	4.91±1.07^*^	1.39±0.89	1.40±0.38	2.92±0.90^**^	1.42±0.20	0.98±0.27^*^	1.52±0.63^*^
**ε2/ε4**	27	4.73±0.98^*^	1.54±0.87	1.33±0.40	2.83±0.79^*^	1.39±0.16	1.10±0.30	1.29±0.32
**ε3/ε3**	989	5.16±0.99	1.43±0.97	1.35±0.34	3.29±0.87	1.38±0.18	1.04±0.27	1.36±0.38
**ε3/ε4**	199	5.19±1.14	1.59±1.15	1.35±0.56	3.27±0.99	1.36±0.11	1.07±0.42	1.26±0.54
**ε4/ε4**	17	5.33±0.96	1.44±0.87	1.36±0.33	3.38±0.78	1.38±0.09	1.22±0.23	1.29±0.26

Data are expressed as the mean ± SD. The ε2 carriers included the genotypes ε2/ε2, ε2/ε3 and ε2/ε4; the ε3 carriers included ε3/ε3; the ε4 carriers included ε3/ε4 and ε4/ε4. *TC* Total cholesterol, TG=Triglyceride, *HDL-C* High density lipoprotein cholesterol, *LDL-C* Low density lipoprotein cholesterol. *ApoA1* apolipoproteinA1, *ApoB* apolipoprotein B. A1/B, the ratio of ApoA1 and ApoB

Continuous variables were compared using unadjusted one-way ANOVA, LSD (L) post-test were performed to determine the differences in lipid parameters among different genotypes or alleles

*P<0.05, **P<0.01, ε2 carriers VS noncarriers (ε3 carriers and ε4 carriers); ^#^*P*<0.05,ε2/ε2 genotype VS ε2/ε3 and ε2/ε4 genotype

Although the serum TC of the ε4 carriers was higher than the ε3 carriers intuitively, there were no significant differences in the carriers of ε4 compared to the ε3 carriers (*P*>0.05, Fig. 1A).

Based on the APOE genotype analysis, the serum TC, LDL-C, and ApoB were also lower in the ε2/ε2, ε2/ε3, and ε2/ε4 genotypes when compared with ε3/ε3, ε3/ε4, and ε4/ε4 (all *P* < 0.05; Table 3; Fig. 1C and D). Interestingly, the serum LDL-C and ApoB in the ε2/ε2 genotype were lower than the ε2/ε3 and ε2/ε4 genotypes, while serum A1/B in the ε2/ε2 genotype was higher than the ε2/ε3 and ε2/ε4 genotypes. However, no significant differences were observed when compared to the ε3/ε4 and ε4/ε4 genotype (all *P* > 0.05; Table 3; Fig. 1C and D). These results suggest that the effects of APOE on serum lipids were gene-dependent, particularly in the ε2 carriers. The influence of the APOE genotype in serum lipids was consistent with the APOE carriers.

### Serum lipids according to gender in different APOE carriers

**Fig. 2:**
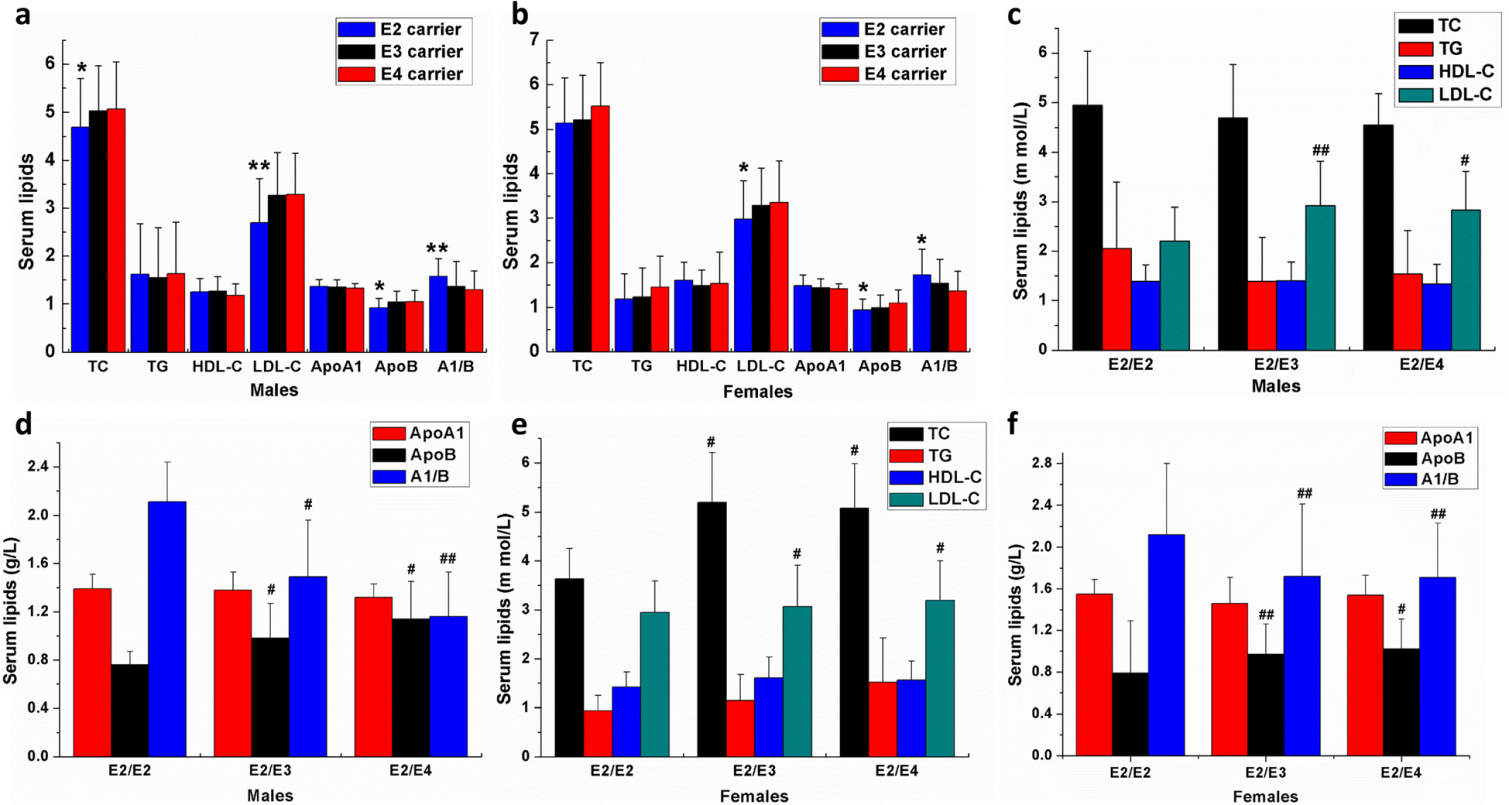
The effects of APOE genetic polymorphisms on serum lipids of the middle-aged and elderly Chinese Fujian Han population in males and females allele carriers. The effects of APOE allele carriers on serum lipids of the middle-aged and elderly Fujian Han population in males (**A**) and females (**B**); The effects of the genotypes of APOE ε2 carriers (ε2/ε2, ε2/ε3 and ε2/ε4) on serum lipids of the middle-aged and elderly Fujian Han population in males (**C** and **D**) and females (**E** and **F**). TC=Total cholesterol, TG=Triglyceride, HDL-C=High density lipoprotein cholesterol, LDL-C=Low density lipoprotein cholesterol. ApoA1=apolipoproteinA1, ApoB=apolipoprotein B. *P<0.05, **P<0.01, VS noncarrier (ε3 carrier and ε4 carrier)

To evaluate the effects of APOE on the serum lipids in the gender, we analyzed the data of serum lipids in males and females, respectively. As shown in Table 4, in males, the serum TC, LDL-C, and ApoB of the ε2 carriers were lower than those of ε3 and ε4. The serum A1/B in the ε2 carriers was higher than the carriers of ε3 and ε4 (all *P* < 0.05, Fig. 2A), while there were no significant differences between the ε3 and ε4 carriers (all *P* > 0.05, Fig. 2A). At the same time, in females, the serum LDL-C and ApoB of ε2 carriers were lower than those of the ε3 and ε4 carriers. In contrast, serum A1/B in the ε2 carriers was higher than the carriers of ε3 and ε4 (all *P* < 0.05; Fig. 2B). The serum TC of the ε4 carriers was higher than the ε3 carriers and the ε2 carriers intuitively; however, there were no significant differences (all *P* > 0.05; Fig. 2B), which were different from the results in all subjects and the males (Fig. 2A).

**Table 4:** The influence of Alleles of APOE and sex in serum lipid profile in middle-aged and elderly Fujian Han population

Allele	Male (*n*=795)			*P* value	Female (*n*=624)			*P* value
	**ε2 (115)**	**ε3(563)**	**ε4(117)**		**ε2(99)**	**ε3(426)**	**ε4(99)**	
**TC**	4.69±1.01*	5.03±0.94	5.07±0.98	0.030	5.14±1.01	5.22±0.99	5.53±0.97	0.172
**TG**	1.62±1.05	1.55±1.04	1.63±1.08	0.853	1.18±0.57	1.23±0.65	1.45±0.70	0.114
**HDL-C**	1.25±0.28	1.27±0.30	1.18±0.24	0.182	1.60±0.41	1.48±0.35	1.53±0.71	0.363
**LDL-C**	2.70±0.92^**^	3.27±0.89	3.29±0.86	<0.001	2.98±0.86^*^	3.29±0.84	3.35±0.94	0.037
**ApoA1**	1.37±0.14	1.35±0.15	1.33±0.10	0.278	1.48±0.24	1.43±0.20	1.41±0.11	0.206
**ApoB**	0.93±0.19^*^	1.04±0.23	1.05±0.23	0.024	0.94±0.24^*^	0.99±0.28	1.09±0.29	0.015
**A1/B**	1.58±0.37^*^	1.37±0.52	1.30±0.39	0.038	1.72±0.58^*^	1.54±0.53	1.36±0.45	0.029
**Genotype**	**ε2/ε2 (7)**	**ε2/ε3(92)**	**ε2/ε4(16)**		**ε2/ε2(4)**	**ε2/ε3(84)**	**ε2/ε4(11)**	
**TC**	4.95±1.09	4.70±1.07	4.55±0.63	0.315	3.64±0.62	5.19±1.02^#^	5.08±0.91^#^	0.038
**TG**	1.67±1.31	1.59±1.04	1.55±0.92	0.202	0.94±0.31	1.14±0.53	1.52±0.90	0.156
**HDL-C**	1.31±0.36	1.26±0.26	1.22±0.37	0.436	1.42±0.31	1.61±0.42	1.56±0.39	0.765
**LDL-C**	1.77±0.33	2.81±0.94^##^	2.67±0.49^#^	<0.001	2.95±0.64	3.07±0.84^#^	3.19±0.81^#^	0.036
**ApoA1**	1.39±0.12	1.38±0.15	1.32±0.11	0.590	1.55±0.14	1.46±0.25	1.54±0.19	0.491
**ApoB**	0.76±0.11	0.98±0.29^#^	1.14±0.31^#^	0.038	0.79±0.50	0.97±0.29^##^	1.02±0.29^#^	0.002
**A1/B**	2.11±0.33	1.49±0.67^#^	1.16±0.37^##^	0.043	2.12±0.68	1.72±0.69^##^	1.71±0.52^##^	<0.001

Data are expressed as the mean ± SD. *TC* Total cholesterol, *TG* Triglyceride, *HDL-C* High density lipoprotein cholesterol, *LDL-C* Low density lipoprotein cholesterol. *ApoA1* apolipoproteinA1, *ApoB* apolipoprotein B. A1/B, the ratio of ApoA1 and ApoB. Values were compared using unadjusted one-way ANOVA, LSD (L) post-test were performed to compare the differences in lipid parameters among different carriers

*P<0.05, **P<0.01, ε2 carriers VS noncarriers (ε3 carriers and ε4 carriers); ^#^*P*<0.05,^##^*P*<0.01, compared with the ε2/ε2 genotype

We further evaluated the effects of the genotype on serum lipids. As shown in Table 4, the serum LDL-C and ApoB in the ε2/ε2 genotype were significantly lower, and the serum A1/B was higher in the ε2/ε2 genotype, both in males (Fig. 2C and D) and females (Fig. 2E and F; all *P* < 0.05) when compared with the genotype of ε2/ε3 and ε2/ε4. However, the serum TC in the ε2/ε2 genotype was no different compared with the genotype of ε2/ε3 and ε2/ε4 in males (*P >* 0.05, Fig. 2C), while the serum TC in the ε2/ε2 genotype was lower than the ε2/ε3 and ε2/ε4 genotypes in females (all *P >* 0.05, Fig. 2E). Additionally, no significant differences were observed in the genotype of ε3/ε4 and ε4/ε4 (data not shown). These data indicated that the influence of the APOE on serum lipids was gender-dependent, especially in the ε2 allele. Therefore, we only analyzed the genotype of ε2/ε2, ε2/ε3, and ε2/ε4 in the following study.

### Serum lipids according to the carriers of APOE allele, age, and gender

**Fig. 3:**
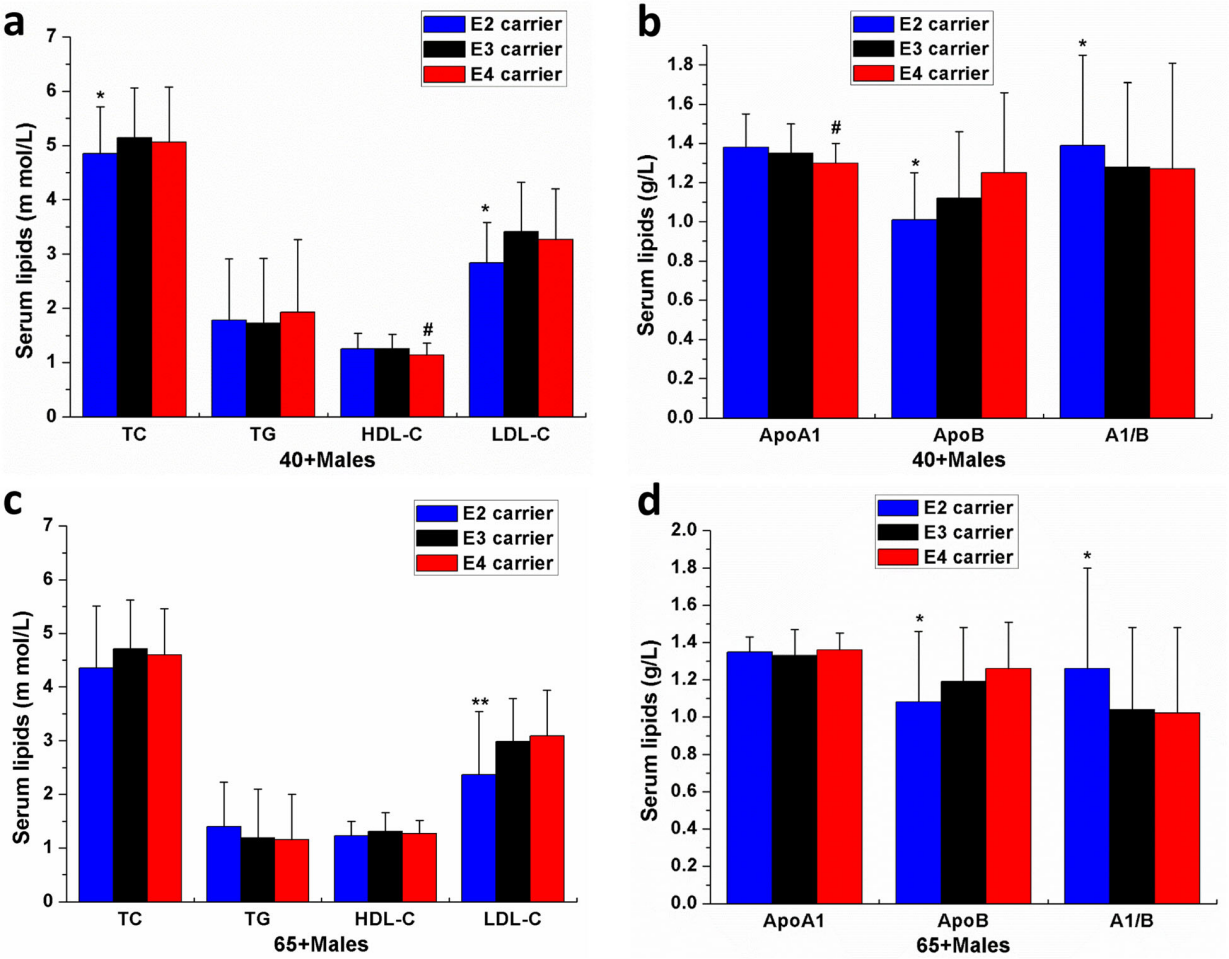
The effects of APOE allele carriers and age on serum lipids in the middle-aged (40+) and elderly (65+) males of Fujian Han population. The influence of APOE allele carriers and age on serum TC, TG, HDL-C and LDL-C (**A**) and serum ApoA1, ApoB and A1/B (**B**) in the 40+ males; The influence of APOE allele carriers and age on serum TC, TG, HDL-C and LDL-C (**C**) and serum ApoA1, ApoB and A1/B (**D**) in the 65+ males. TC=Total cholesterol, TG=Triglyceride, HDL-C=High density lipoprotein cholesterol, LDL-C=Low density lipoprotein cholesterol. ApoA1=apolipoproteinA1, ApoB=apolipoprotein B. *P<0.05, **P<0.01, ε2 carriers VS noncarriers (ε3 carriers sand ε4 carriers); ^#^*P*<0.05, ε4 carriers VS noncarriers (ε3 carriers and ε2 carriers)

Based on the aforementioned results, to further evaluate the combined effects of the APOE, gender, and age on serum lipids, we divided the subjects into the middle-aged group (age from 40 to 64 years old, referred to as 40+) and the elderly group (age ≥ 65 years old, referred to as 65+) for both males and females. As shown in Table 5, in 40+ males, the serum TC, LDL-C, and ApoB in the APOE ε2 carriers were lower than in the carriers of ε3 and ε4, and serum A1/B in the ε2 carriers was higher than the noncarriers (all *P* < 0.05; Fig. 3A and B). However, the serum HDL-C and ApoA1 in the ε4 carriers were lower than the ε2 and ε3 carriers (all *P* < 0.05; Fig. 3A and B) in 40+ males, which was not observed in whole males and all participants. Then, in the 65+ males, the serum LDL-C and ApoB in the ε2 carriers were lower than in the noncarriers, and serum A1/B was higher in the ε2 carriers (all *P* < 0.05, Fig. 3C and D). However, the serum TC of the ε2 carriers showed no significant differences compared with the ε3 carriers (*P* > 0.05, Fig. 3C), which was inconsistent with the 40+ males and the whole males.

**Table 5:** The effects of APOE, age, and sex in serum lipid profile in middle-aged and elderly Fujian Han population

serum lipids	Male (795)				Female (624)			
	**all**	**ε2 (115)**	**ε3(563)**	**ε4(117)**	**all**	**ε2 (99)**	**ε3(426)**	**ε4(99)**
**40-64**	*N*=481	*N*=65	*N*=350	*N*=66	*N*=355	*N*=54	*N*=244	*N*=57
**TC**	5.09±0.93	4.85±0.86^*^	5.14±0.92	5.06±1.02	5.20±0.99	5.12±0.97	5.19±1.02	5.33±0.95
**TG**	1.75±1.25	1.78±1.13	1.72±1.20	1.93±1.33	1.19±0.65	1.14±0.57	1.16±0.71	1.34±0.45
**HDL-C**	1.23±0.27	1.25±0.29	1.25±0.27	1.14±0.22^#^	1.52±0.36	1.65±0.42^*^	1.49±0.33	1.53±0.41
**LDL-C**	3.31±0.91	2.84±0.74^*^	3.41±0.91	3.26±0.94	3.23±0.86	3.01±0.78	3.25±0.87	3.34±0.88
**ApoA1**	1.35±0.15	1.38±0.17	1.35±0.15	1.30±0.10^#^	1.44±0.20	1.52±0.26^*^	1.42±0.21	1.43±0.11
**ApoB**	1.12±0.38	1.01±0.24^*^	1.12±0.34	1.25±0.61	1.05±0.28	0.98±0.28	1.04±0.28	1.07±0.28
**A1/B**	1.29±0.46	1.39±0.46^*^	1.28±0.43	1.27±0.64	1.44±0.54	1.66±0.46^*^	1.44±0.52	1.32±0.48
**65+**	*N*=314	*N*=50	*N*=213	*N*=51	*N*=269	*N*=45	*N*=182	*N*=42
**TC**	4.63±0.95	4.35±1.16	4.71±0.91	4.60±0.86	5.30±1.11	5.17±1.12	5.32±0.95	5.33±1.02
**TG**	1.22±0.83	1.40±0.83	1.19±0.91	1.15±0.85	1.46±0.72	1.24±0.59	1.38±0.53	1.82±0.84^#^
**HDL-C**	1.29±0.33	1.23±0.27	1.31±0.35	1.27±0.24	1.48±0.59	1.49±0.38	1.47±0.42	1.53±0.61
**LDL-C**	2.90±0.88	2.36±1.17^**^	2.99±0.79	3.09±0.85	3.34±0.98	3.09±0.94	3.39±0.77	3.38±0.88
**ApoA1**	1.34±0.12	1.35±0.08	1.33±0.14	1.36±0.09	1.41±0.18	1.40±0.15	1.43±0.19	1.37±0.12
**ApoB**	1.19±0.31	1.08±0.38^*^	1.19±0.29	1.26±0.25	1.22±0.29	1.17±0.41	1.22±0.25	1.27±0.32
**A1/B**	1.03±0.68	1.26±0.54^*^	1.04±0.64	1.02±0.46	1.11±0.59	1.11±0.45	1.12±0.50	1.08±0.38

Data are expressed as the mean ± SD.*TC* Total cholesterol, *TG* Triglyceride, *HDL-C* High density lipoprotein cholesterol, *LDL-C* Low density lipoprotein cholesterol. *ApoA1* apolipoproteinA, *ApoB* apolipoprotein B. A1/B, the ratio of ApoA1 and ApoB. Values were compared using unadjusted one-way ANOVA, LSD (L) post-test were performed to determine the differences in lipid parameters among different allele carriers

*P<0.05, **P<0.01, ε2 carriers VS noncarriers (ε3 carriers and ε4 carriers); ^#^*P*<0.05, ε4 carriers VS noncarriers (ε3 carriers and ε2 carriers)

**Fig. 4:**
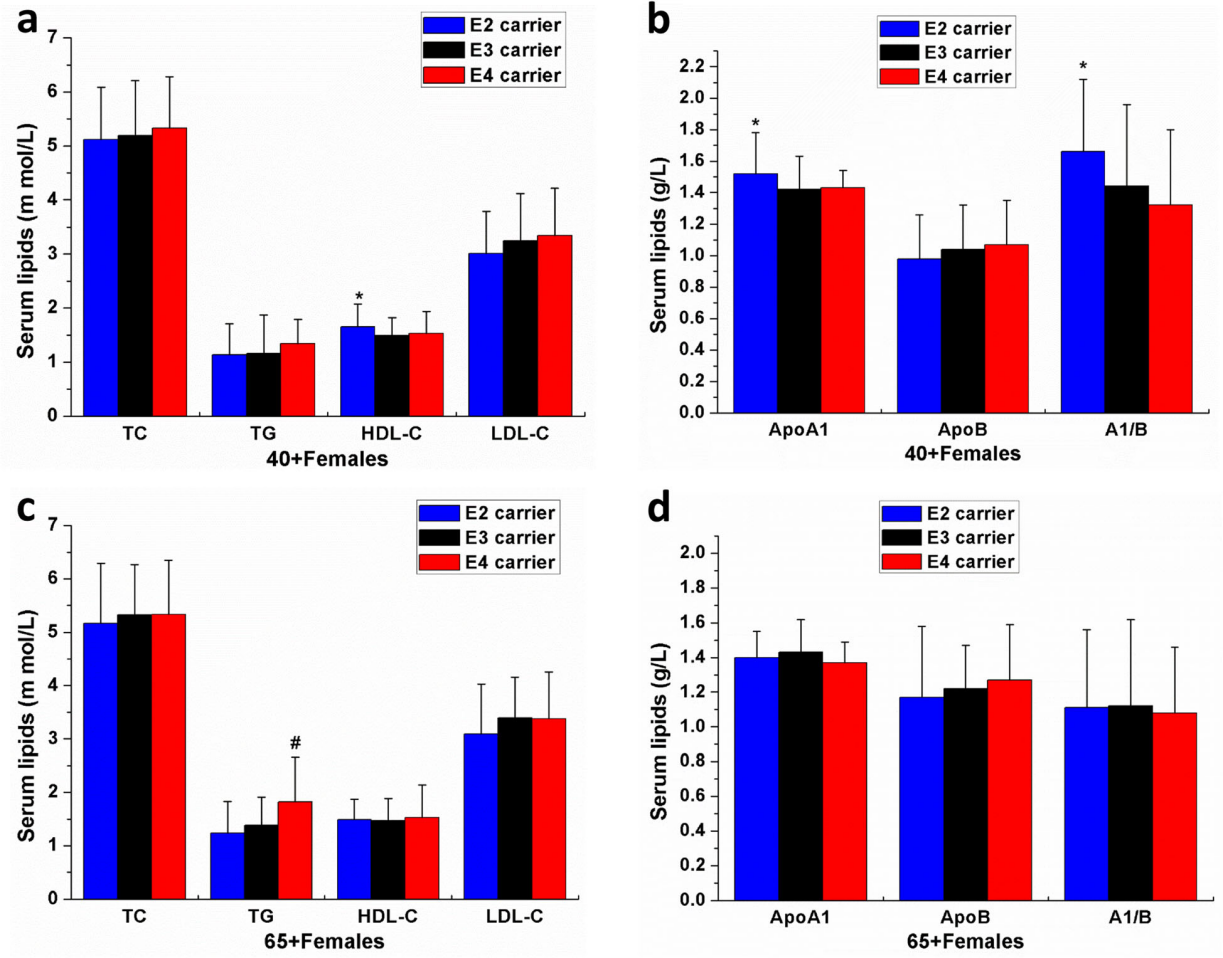
The effects of APOE allele carriers and age on serum lipids in the middle-aged (40+) and elderly (65+) females of Fujian Han population. The influence of APOE allele carriers and age on serum TC, TG, HDL-C and LDL-C (**A**) and serum ApoA1, ApoB and A1/B (**B**) in the 40+ females; The influence of APOE allele carriers and age on serum TC, TG, HDL-C and LDL-C (**C**) and serum ApoA1, ApoB and A1/B (**D**) in the 65+ females. TC=Total cholesterol, TG=Triglyceride, HDL-C=High density lipoprotein cholesterol, LDL-C=Low density lipoprotein cholesterol. ApoA1=apolipoproteinA1, ApoB=apolipoprotein B. *P<0.05, **P<0.01, ε2 carriers VS noncarriers (ε3 carriers and ε4 carriers); ^#^*P*<0.05, ε4 carriers VS noncarriers (ε3 carriers and ε2 carriers)

Meanwhile, in 40+ females, the serum HDL-C and ApoA1 of the ε2 carriers were higher than the noncarriers (all *P* < 0.05; Fig. 4A and B). Then, in 65+ females, only the serum TG in the ε4 carriers was higher than the noncarriers (*P* < 0.05; Fig. 4C and D). In contrast, no significant difference was found in the serum TC, LDL-C, and ApoB among different APOE allele carriers in 40+ females and 65+ females (all *P* > 0.05; Fig. 4C and D). These results indicated that the effects of the APOE allele carriers on serum lipids were also in an age- and gender-dependent manner both in males and females.

### The association of APOE allele carrier status and at-risk levels of dyslipidemia

To estimate the associations between the APOE allele carrier status and the at-risk levels of dyslipidemia in the middle-aged and elderly population, we used the logistic regression analysis to determine the odds ratios (OR), which reveal the at-risk levels of dyslipidemia. The diagnosis of dyslipidemia was based on the Guidelines for the Prevention and Treatment of Dyslipidemia in Chinese Adults (revised 2016) [21].

As shown in Table 6, for 40+ males and 65+ males, after adjusting for BMI, education, physical activity, smoking, and alcohol drinking, the APOE ε2 carriers were less likely to have at-risk levels of high LDL-C (≥ 3.4 m mol/L) and the value of OR was 0.165 (95 % CI = 0.061-0.444, *P* = 0.001) and 0.204 (95 % CI = 0.060-0.916, *P* = 0.034), respectively. Later, in 40+ females, the ε2 carriers were less likely to have at-risk levels of low HDL-C (< 1.0 m mol/L), while the others had no significant differences. Particularly in 65+ females, the ε4 carriers were more likely to have at-risk levels of high TG (≥ 1.7 m mol/L). The value of OR was 2.937 (95 % CI = 1.026-8.464, *P* = 0.046).

**Table 6:** Odds ratios from logistic regression models predicting at-risk levels of serum lipids by APOE ε2 and ε4 carriers in middle-aged (40+) and elderly (60+) males or females

	Serum	ε2 carrier		ε4 carrier	
	**Outcome**	***P*value**	**OR (95% CI)**	***P*value**	**OR (95% CI)**
**Male**	**TC (≥5.2)**	0.080	0.494(0.224,1.088)	0.701	0.824(0.411,1.817)
**40+**	**TG (≥1.7)**	0.806	0.909(0.425,1.945)	0.431	0.726(0.327,1.612)
	**HDLC (<1.0)**	0.878	1.078(0.414,2.805)	0.065	0.466(0.207,1.057)
	**LDL-C (≥3.4)**	0.001^**^	0.165(0.061,0.444)	0.112	0.539(0.252,1.155)
**65+**	**TC (≥5.2)**	0.608	0.730(0.220,2.430)	0.702	0.806(0.267,2.434)
	**TG (≥1.7)**	0.072	3.556(0.894,14.135)	0.346	2.113(0.217,5.711)
	**HDLC (<1.0)**	0.210	3.303(0.471,10.723)	0.658	1.357(0.445,10.020)
	**LDL-C (≥3.4)**	0.034^*^	0.204(0.060,0.916)	0.953	0.969(0.339,2.768)
**Female**	**TC (≥5.2)**	0.985	1.009(0.424,2.401)	0.664	0.836(0.371,1.880)
**40+**	**TG (≥1.7)**	0.374	0.386(0.048,3.139)	0.290	1.855 (0.591,5.822)
	**HDLC (<1.0)**	0.998	0(----, ----)	0.601	0.523(0.046,5.965)
	**LDL-C (≥3.4)**	0.109	0.598(0.339,1.551)	0.746	1.143(0.509,2.564)
**65+**	**TC (≥5.2)**	0.954	1.037(0.297,3.616)	0.944	1.036(0.373,2.886)
	**TG (≥1.7)**	0.602	0.646 (0.125,3.333)	0.046^*^	2.937(1.026,8.464)
	**HDLC (<1.0)**	0.203	0.288(0.043,1.954)	0.195	0.327(0.060,1.774)
	**LDL-C (≥3.4)**	0.668	0.750(0.201,2.796)	0.698	1.227(0.437,3.448)

*OR* odds ratio; *CI* confidence interval. The confounding factors including age, BMI, education, smoking, alcohol drinking, and physical activity were critically adjusted. *TC* Total cholesterol, *TG* Triglyceride, *HDL-C* High density lipoprotein cholesterol, *LDL-C* Low density lipoprotein cholesterol. *ApoA1* apolipoproteinA1, *ApoB* apolipoprotein B

The diagnostic criteria for dyslipidemia: high TC (≥5.2 m mol/L), high TG (≥1.7 m mol/L), high LDL-C (≥3.4 m mol/L), and low HDL-C (<1.0 m mol/L)

*P<0.05, **P<0.01, VS ε3 carriers

## Discussion

This study focused on the combined effects of APOE genetic polymorphism, age, and sex on serum lipids during aging. Our results showed that the influences of APOE genetic polymorphisms on the serum lipid profiles during aging were both gender- and age-dependent. The carrier of APOE ε2 could be a protective factor of dyslipidemia, while APOE ε4 carriers might be closely associated with the high TG, particularly in elderly females.

First, our results showed that the genotypes and allelic distribution of APOE in the middle-aged and elderly Chinese Fujian Han population were no different compared with the previously reported results in the Asian population [19, 22]. Then, we preliminarily evaluated the influences of the APOE genetic polymorphisms on serum lipids. Our results indicated that the serum TC, LDL-C, and ApoB of the APOE ε2 carriers were lower than the ε3 carriers. At the same time, there were no significant differences in the ε4 carriers compared with the ε3 carriers.

Meanwhile, the serum TC, LDL-C, and ApoB in the genotypes of ε2/ε2, ε2/ε3, and ε2/ε4 were also lower when compared with ε3/ε3, ε3/ε4, and ε4/ε4. More interesting, the serum LDL-C and ApoB in the ε2/ε2 genotype were lower, while serum A1/B in the ε2/ε2 genotype was higher compared with the ε2/ε3 and ε2/ε4 genotypes. However, no significant differences were observed between the ε3/ε4 and ε4/ε4 genotypes.

The gender differences in serum lipids during aging have been reported in many pieces of literature [12]. The present study showed that the serum TC, LDL-C, and ApoB of the ε2 carriers were lower than that of the ε3 and ε4 carriers in males. In females, the serum LDL-C and ApoB in the ε2 carriers were also lower than the carriers of ε3 and ε4, while the serum TC showed no significant differences. Then, the effects of the genotype on serum lipids were also evaluated. The significant differences were only observed in the genotype of ε2 carriers. The serum LDL-C and ApoB in the ε2/ε2 genotype were significantly lower, and the serum A1/B was higher in the ε2/ε2 genotype, both in males and females. While the serum TC in the ε2/ε2 genotype was lower than the ε2/ε3 and ε2/ε4 genotypes in females. These data indicated that the influence of the APOE on serum lipids was gender-dependent, particularly in the ε2 allele.

Next, we observed the differences in serum lipids between middle-aged (40+) and the elderly (65+) males and females, respectively. The serum LDL-C and ApoB in ε2 carriers were significantly lower than the noncarriers, both in the 40+ males and 65+ males. However, the serum HDL-C of ApoA1 for 40+ males in ε4 carriers was substantially lower than the ε2 and ε3 carriers, and the serum TC for 65+ males in ε2 carriers had no significant differences compared to noncarriers. Meanwhile, the serum HDL-C and ApoA1 for 40+ females in the ε2 carriers were higher than those of ε3 and ε4. Moreover, in 65+ females, the serum TG in the ε4 carriers was higher than in the noncarriers, while the other serum lipids showed no significant differences. These results indicated that the effects of the APOE allele carriers on serum lipids were gender- and age-dependent for the middle-aged (40+) and the elderly (65+) populations.

Finally, according to the 2016 China dyslipidemia criteria, using the logistic regression models, we found that, for 40+ males and 65+ males, the APOE ε2 carriers were less likely to have at-risk levels of high LDL-C (≥ 3.4 m mol/L). Meanwhile, in 40+ females, the carriers of APOE ε2 were less likely to have at-risk levels of low HDL-C (< 1.0 m mol/L). Interestingly, in 65+ females, the carriers of APOE ε4 were more likely to have at-risk levels of high TG (≥ 1.7 m mol/L).

The role of the APOE genetic polymorphism in the serum LDL-C in the general population has been reported in many studies, and the APOE ε2 demonstrated lower serum LDL-C levels [23–25]. In this study, the serum LDL-C and ApoB in the ε2 carriers were significantly lower than non-ε2 carriers in middle-aged (40+) and elderly (65+) males. Still, there were no significant differences in middle-aged (40+) females. This result was consistent with the aforementioned previous studies [9, 26]. The association of the ε4 carriers with the serum TC is inconsistent at present. This study is consistent with those studies that observed no significant difference in the ε4 carriers [11, 22]. While the other reported that serum TC in the ε4 carriers was higher compared to noncarriers [27]. The inconsistency may be attributed to differences in the study population, race, geographical area, and study size. The serum TC in the ε2 carriers was lower than noncarriers in 40+ males. However, there were no differences in 65+ males and 40+ females. The concentrations of serum HDL-C and ApoA1 of ε4 carriers were lower than non-ε4 carriers in 40+ males, while in 40+ females the serum HDL-C and ApoA1 were higher in the ε2 carriers compared to the noncarriers.

Notably, the serum TG in the ε4 carriers was significantly higher than the noncarriers (ε2 and ε3) in elderly (65+) females, which was partly consistent with the other study [28]. The differences between various studies might be related to the study population, such as race and dietary habits. Significantly, serum TG is closely associated with diet [29]. The Fujian Province is located on the southeast coast of China. Residents of the Fujian Province love to eat seafood and aquatic products, particularly soup, referred to as “Fujian cuisine,” characterized by low salt and low fat. Meanwhile, most of them have a habit of drinking tea.

The molecular mechanisms of difference of serum lipids among APOE carriers remain unclear; however, they might be related to the structural differences among ApoE2, ApoE3, and ApoE4 [25]. The APOE ε2 carriers display low LDL-C levels that may be associated with the defective binding of ApoE2 to LDL receptors (LDLR) [30]. Studies have suggested the ApoE3 and ApoE4 bind to the LDLR with similar affinity, but the binding of ApoE2 to LDLR is poor, which decreases the hepatic uptake of VLDL [31]. As a consequence, APOE ε2 carriers have lower levels of LDL-C. However, the exaggerated TG response in APOE ε4 carriers may be related to the different lipoprotein distributions of ApoE3 and ApoE4 in serum, thereby affecting the metabolic rate of ApoE-containing lipoproteins [32]. Studies have shown that compared with ApoE3, ApoE4 binds better to VLDL because of its higher lipid affinity [33]. After secretion from the liver into the plasma, TG in VLDL is hydrolyzed by lipoprotein lipase, leading to the creation of intermediate density lipoprotein (IDL); however, excess of ApoE4 on the surface of VLDL particles can impair their lipolysis, which elevates plasma TG levels [34]. Further research will be needed to discover the exact mechanisms of the combined effects of APOE, sex, and age on serum lipids.

### Study strength and limitations

To our knowledge, this is the first population-based association study to explore the combined effects of APOE genetic polymorphisms, gender, and age on serum lipids in the middle-aged and elderly Chinese Fujian Han population. Our results could provide robust evidence for preventing and managing dyslipidemia in China’s middle-aged and elderly population, particularly for individualized prevention based on the APOE genotypes.

However, some limitations should also be noted. First, the variations from the study of individuals should be considered. Many factors could contribute to the serum lipid levels, particularly dietary factors, lifestyle, and random factors [35]. Meanwhile, the serum hormonal levels are different in the middle-aged and elderly population, particularly for females. When we estimate the effects of gender on serum lipids, serum hormonal levels should be considered. If we can provide the serum hormonal levels in the current study, the evidence of gender on serum lipids would be more powerful. Moreover, the original aim of this study was to identify the risk factors or individuals based on the APOE genotypes. However, the final data showed the carrier of APOE ε2 could be a protective factor of dyslipidemia, while only in the elderly (65+) females, the APOE ε4 carriers might be a risk factor for high TG, which might limit the innovation of this study to a certain extent. Finally, our study was performed in a single center, which would limit the ability to draw major conclusions on all populations.

## Conclusions

In conclusion, the effects of the genetic polymorphisms of APOE on the serum lipids were both gender- and age-dependent in the middle-aged and elderly Chinese Fujian Han population. Our results could provide useful evidence for preventing and managing dyslipidemia via personalized strategies in China’s middle-aged and elderly population, particularly based on the APOE genotype.

## Data Availability

Not applicable.
